# Mycobacterial lipoarabinomannan negatively interferes with macrophage responses to *Aspergillus fumigatus in-vitro*

**DOI:** 10.1371/journal.pone.0351935

**Published:** 2026-07-09

**Authors:** Luis E. Gonzales-Huerta, Thomas J. Williams, Renad Aljohani, Brian Robertson, Carlton A. Evans, Darius P. H. Armstrong-James

**Affiliations:** 1 Department of Infectious Disease, Imperial College London, London, United Kingdom; 2 Departamento de Investigación, Instituto de Medicina Traslacional, Lima, Peru; 3 Innovación Por la Salud Y Desarrollo (IPSYD), Asociación Benéfica PRISMA, Lima, Peru; 4 IFHAD: Innovation For Health And Development, Laboratorio de Investigación y Desarrollo, Universidad Peruana Cayetano Heredia, Lima, Peru; 5 IFHAD: Innovation For Health And Development, Department of infectious disease, Imperial College London, London, United Kingdom; Gulu University, UGANDA

## Abstract

Over 1 million people have chronic pulmonary aspergillosis (CPA) secondary to pulmonary tuberculosis. Additionally, *Aspergillus fumigatus* (*Af*) has been reported as one of the most common pathogens associated with mycobacteria in patients with cystic fibrosis. Mycobacterial virulence factors, like lipoarabinomannan, have been shown to interfere with host’s intracellular pathways required for an effective immune response, however, the immunological basis for mycobacterial-fungal coinfection is still unknown. We therefore investigated the effect of lipoarabinomannan on macrophage responses against *Af*. Bone marrow-derived macrophages (BMDMs) were stimulated with non-mannose-capped lipoarabinomannan (LAM) from *Mycobacterium smegmatis* or mannose-capped lipoarabinomannan (ManLAM) from *Mycobacterium tuberculosis* for 2 hours and then infected with swollen *Af* conidia. Cell death was assessed by lactate dehydrogenase release. Cytokine release was measured in supernatant using Enzyme Linked Immuno-Sorbent Assay (ELISA). Colony forming units counting and time-lapse fluorescence microscopy was performed for studying conidia killing by macrophages. BMDMs stimulated with LAM showed increased cell death and inflammatory cytokine release in a dose-dependent manner, characterised by a significant increase of IL-1β release. Time-lapse fluorescence microscopy and CFUs revealed that both LAM and ManLAM significantly decrease the capacity of macrophages to kill *Af* conidia within the first 6 hours of infection. The mycobacterial virulence factor, lipoarabinomannan, disrupts macrophage capacity to efficiently clear *Af* at early stages of infection *in-vitro*.

## Introduction

*Aspergillus fumigatus* (*Af*) is a ubiquitous airborne fungus, and approximately 100–1000 conidia are believed to reach the lower respiratory tract and alveoli each day. Immunocompetent hosts clear fungi in the airway with effective innate immune responses [1]. However, immunocompromised individuals, such as patients with neutropenia, cancer, lung transplant recipients, or with high exposures to corticosteroids, are susceptible to develop pulmonary aspergillosis (PA) [2]. Additionally, patients with conditions that cause structural damage to the airway and pulmonary parenchyma, such as cystic fibrosis (CF), sarcoidosis, and lung cavitation post-pulmonary tuberculosis, present a higher risk for PA [3], which has an estimated 5-year mortality rate between 50% and 85% [4]. In patients with CF, the association between mycobacteria and *Af* indicates increased mortality [5–7]. Furthermore, the burden of PA post-tuberculosis is considered to be substantial [8], however, the causative factors for a close association between these pathogens are still unknown.

The alveolar macrophages are considered the first line of defence against *Af*. In the alveoli, macrophages take over the task of recognizing and process *Af* conidia. A protective rodlet protein layer in conidia covers the pathogen-associated molecular pattern (PAMPs) from pattern recognition receptor (PRRs) in immune cells. However, lung tissue and the body temperature of the host provide the necessary conditions for swelling and germination to initiate. The conidia swelling breaks the rodlet layer and exteriorizes galactomannan, chitin, α-glucan, and β-glucan. These components will bind to a diversity of receptors in the macrophage, inducing a mechanism that will kill conidia or inhibit their germination [9]. Recognition of β-glucan by TLR-2, induces the plasma membrane receptor to activate the myeloid differentiation primary response 88 (MyD88) downstream to NF-κβ [10]. Similarly, TLR-4 recognizes mannan, contributing to MyD88-mediated signalling pathway. Both, lead to increased expression and release of inflammatory cytokines, TNF-α and IL-12 [11]. Additionally, C-type lectin receptors (CTL) recognize carbohydrate ligands, intervening in phagocytosis, and collaborating in the release of inflammatory cytokines [12,13]. Additionally, Dectin-1 is crucial in the phagocytosis process of conidia, by recognizing β-glucans in *Af*, it activates the immunoreceptor tyrosine-based activation motif (ITAM). This leads to subsequent triggering of Src/Syk-dependent signalling pathway to contribute in the activation of NF-κβ and NFAT [14,15]. Mannose receptor recognizes chitin, internalize it, and has been shown to modulate macrophage activation by increasing the production of IL-10, which dampens the inflammation [16].

NLRs have been also associated with the immune response against *Af*. Although, NOD2 expression contributes to the inflammatory response through activation of NF-κβ in *Af* infection [17], the genetic deficiency of NOD2 has been shown to be protective against invasive aspergillosis [18]. NLRP3 has been reported to respond effectively to hyphal fragments by triggering the inflammasome assembly, with subsequent caspase-1 activation and IL-1β release, which requires K^+^ efflux and ROS production [19]. Muramyl dipeptide (MDP) is a well-known bacterial ligand for NOD2 [20], but it is absent in *Af*. It has been suggested that inflammasome assembly is triggered by nucleic acid structures released by *Af* [21,22].

Lipoarabinomannan is one of the most important virulence factors in mycobacteria. It is constituted by a polymannosylated phosphatidylinositol core with an arabinan moiety. This arabinan termini can be capped with an inositol phosphate residue or with mannose, depending on the mycobacterial specie, and it modifies its immunogenicity [23]. Mannose-capped lipoarabinomannan (ManLAM) produced by *Mycobacterium tuberculosis*, has been shown to interfere with normal phagolysosome function, inflammatory cytokine production, and to inhibit apoptosis [24–26]. Alternatively, the lipoarabinomannan produced by *Mycobacterium smegmatis*, lacks the mannose cap and has been showed to induce a strong TLR-2 mediated immune response, while it decreases phagocytosis [27].

Epidemiological and clinical data suggest that mycobacteria infection potentially facilitates fungal colonization and respiratory disease. Moreover, mycobacterial lipoarabinomannan has been shown to interfere with the cellular mechanisms that are required for an effective response to *Af*. Therefore, we hypothesize that mycobacterial lipoarabinomannan negatively modulates macrophage responses to *Af*.

## Materials and methods

### Ethics statement

All mouse work was carried out following the Animal [Scientific Procedures] Act 1986. All mouse experiments were approved by the United Kingdom Home Office and the Imperial College London Animal Ethics Committee and performed in accordance with the project license PPL 70/7941.

### Culture and preparation of Aspergillus fumigatus (Af)

*Af* strains CEA-10 (FGSC A1163) was obtained from the Fungal Genetics Stock Center and the American Type Culture Collection (ATCC). A GFP-expressing *Af* derived from ATCC46645 strain (ATCC46645-eGFP) was kindly given by Frank Ebel. A dsRED-expressing *Af* (AF293) was a kind gift from Georgios Chamilos. *Af* was cultured in Sabouraud dextrose agar (Oxiod® Cat. CM0041B) for 3−4 days at 37°C and harvested by washing with DPBS + 0.1% Tween-20. Conidia suspension was filtered using sterile Miracloth™ (Millipore™, Cat. 475855). To generate swollen conidia, 10^6^ conidia per mL of RPMI-1640 (Gibco® Cat. 11875−093) were incubated in petri-dish at 37°C + 5% CO_2_ for 3 hours.

### Tracking of conidia swelling and germination

Resting *Af* conidia was harvested and resuspended at a concentration of 5 x 10^5^ in RPMI-1640. LAM (BEI resources, Cat. NR-14849) and ManLAM (BEI resources NR-14848) were added to the media at a concentration of 1 μg/mL. *Af* conidia were transferred to a 96-well plate (Greiner® Cat. 655090) at a concentration of 10^5^ per well in 200 μL.Conidia were imaged in a widefield inverted microscope (Zeiss® Cell Discoverer 7), with a heated chamber at 37°C and 5% CO_2_. Cells were imaged every 15 min for 12 hours in a single z-plane.Image analysis was performed using FIJI. Nine conidia, properly focused for at least 9 hours, were selected from each FOV. Manual measurements of conidial diameter were taken for every hour of incubation until point of germination was reached.

### Bone marrow-derived macrophages (BMDMs)

Healthy C57BL/6J mice between 8 and 12 weeks old were euthanized with cervical dislocation. Secondary confirmation was performed with dissection of the femoral artery. No invasive procedures or survival interventions were performed before euthanasia, therefore anaesthesia or analgesia were not required. Bone marrow was flushed out of mice femurs with cold PBS and filtered through a 40 μm cell strainer. Cells were centrifuged and resuspended in 60 mL of cRPMI + 40 ng/mL m-CSF (PeproTech® Cat. 315−02) + 50 μg/mL Gentamicin (Gibco®, Cat. 11540506). Cells were cultured in petri-dishes at 37°C + 5% CO_2_. After 3 days, additional 5 mL of fresh media was added, and cells incubated for 4 more days to complete differentiation. To recover BMDMs, medium was discarded, replaced with cold PBS + 2 mM EDTA (Millipore® Cat. 324506−100ML) and petri-dishes placed in the fridge for 20 min. For experimentation, macrophages were seeded in a 96-well flat bottom plate at a concentration of 5 x 10^4^ per well in 200 μL of cRPMI + 20 ng/mL m-CSF. Cells were activated with overnight with 200 U/mL of IFN-γ (Peprotech® Cat. 315−05). On the day of infection, IFN-γ was removed and cells were stimulated with lipoarabinomannan (BEI resources, Cat. NR-14849 and NR-14848) for 2 hours. Cells were infected with CEA-10 *Af* or dsRED-*Af* swollen conidia at MOI = 2.

### LDH assay

BMDMs, were seeded at 5 x 10^4^ cells per well and stimulated for 2 hours with LAM or ManLAM [0.1, 0.5, and 1 μg/mL], prior to infection with CEA-10 Af swollen conidia at MOI = 2. Supernatant was collected 6, 12, 18 or 24 hpi and LDH release was measured using the Cytotox-96® Non-Radioactive Cytotoxicity Assay (Promega® Cat. G1780) according to manufacturer’s instructions. 50 μL of fresh supernatant was employed.

### Cytokine release

BMDMs, were seeded at 5 x 10^4^ cells per well and stimulated for 2 hours with LAM or ManLAM [0.1, 0.5, and 1 μg/mL], prior to infection with CEA-10 Af swollen conidia at MOI = 2. Supernatant was collected 24 hpi.

Cytokines were studied using the supernatant from tissue culture and a sandwich ELISA kit from Bio-Techne for TNF-α (R&D Systems Cat. DY410), IL-1β (R&D Systems Cat. DY401) and CXCL1/KC (R&D Systems Cat. DY453) according to manufacturer’s instructions in Nunc MaxiSorp™ 96-well plates (Invitrogen™, Cat. 44-2404-21).

### Colony forming units (CFUs)

BMDMs, were seeded at 1x10^5^ cells per well were and stimulated in a 24 well plate for experimentation. Cells were infected with CEA-10 swollen conidia at MOI = 2. At 3 hpi, supernatant was removed, cells were washed with warm PBS and lysed with 500 μL of DPBS (Gibco® Cat. 14190–136) + 0.1% Tween-20 (Sigma-Aldrich®, Cat. 9416–100ML). Serial dilutions were prepared from lysed cells. 100 μL of lysed cells dilution (1:100) was transferred and spread on a 9 mm petri-dish containing 15 mL of Sabouraud agar (Oxiod® Cat. CM0041B), incubated at 37°C for 18–24 hours, and counted manually.

### FLARE conidia

Fluorescent Aspergillus Reporter (FLARE) conidia was prepared according to the protocol published by Tobias Hohl’ group [28]. dsRED-*Af* aliquots were centrifuged at 9300 g for 10 min and the supernatant discarded. The pellet is resuspended in 1 mL of 0.05 M NaHCO_3_ pH 8.3 + 500 μg of biotin-XX, SSE (Invitrogen, Cat. 11524197) and incubated at 4°C for 2 hours. Next, *Af* is centrifuged, and pellet resuspended in 1 mL of 100 mM Tris-HCl pH 8.0 for 1 hours at room temperature. Then, *Af* is centrifuged and Tris-HCl discarded. Pellet is resuspended in 1 mL PBS with 20 μg/mL of Streptavidin-AF633 (Invitrogen, Cat. S21375) and incubated for 40 min at room temperature. Conidia is then centrifuged at 9300 g x 10 min, supernatant removed, and pellet resuspended in PBS. For swollen conidia, fungi are counted, new aliquots of 2x10^6^ or 4x10^6^ conidia per mL are prepared with RPMI-1640 (Gibco® Cat. 11875−093) and incubated at 37°C + 5% CO_2_ for 3 hours. Finally, conidia are centrifuged again, medium discarded, and pellet resuspended in culture medium.

### Microscopy tracking of conidia killing assay

Macrophages were seeded in a 96-well (Greiner® Cat. 655090) at a concentration of 5 x 10^4^ per well for BMDMs. On the day of infection, IFN-γ and m-CSF was removed to stimulate the macrophages with 1 μg/mL of LAM, ManLAM, in 100 μL of cRPMI without phenol red. After 1 hour, 100 μL of medium with 100 nM of LysoTracker™ Blue DND-22 (Invitrogen™ Cat. L7525) was added to each condition, preserving the mycobacterial ligands’ concentration. After 2 hours of stimulation, the medium was removed and replaced with 200 μL of fresh cRPMI + ligands. Next, 50 μL of medium with 10^5^ FLARE swollen conidia was added. For controls, 50 μL of medium without *Af* conidia is added to get all condition to 250 μL.

For BMDMs, cells were imaged in an automated high-content widefield inverted microscope (Zeiss® Cell Discoverer 7), with a heated chamber at 37°C and 5% CO_2_. One FOV per well was set for time-lapse images, acquired with Zen Blue 3.1 software in 16-bit. Cells were imaged every 15 min for 6 hours in a single z-plane.

Image analysis was performed using FIJI. Channels for Lysotracker™ Blue DND-22, dsRED and AF633 were split using a FOV from an unstimulated well with *Af* infection to manually determine the threshold for positivity. These threshold values were set for the rest of FOVs. dsRED and AF633 channels were employed for counting conidia killing manually.

### Statistical analysis

All values are shown as biological replicates. All statistical analysis were performed on biological replicates. To determine statistical significance between two unmatched groups Students-T test was used. For significance testing in data normally distributed between more than two groups in one variable, One-way analysis of variance (ANOVA) was applied. Comparisons between two or more groups and two variables were analysed using Two-way ANOVA. Statistical analysis was perform using GraphPad Prism 9.4.1.

## Results

### Lipoarabinomannan does not alter conidial swelling and germination

**Fig 1:**
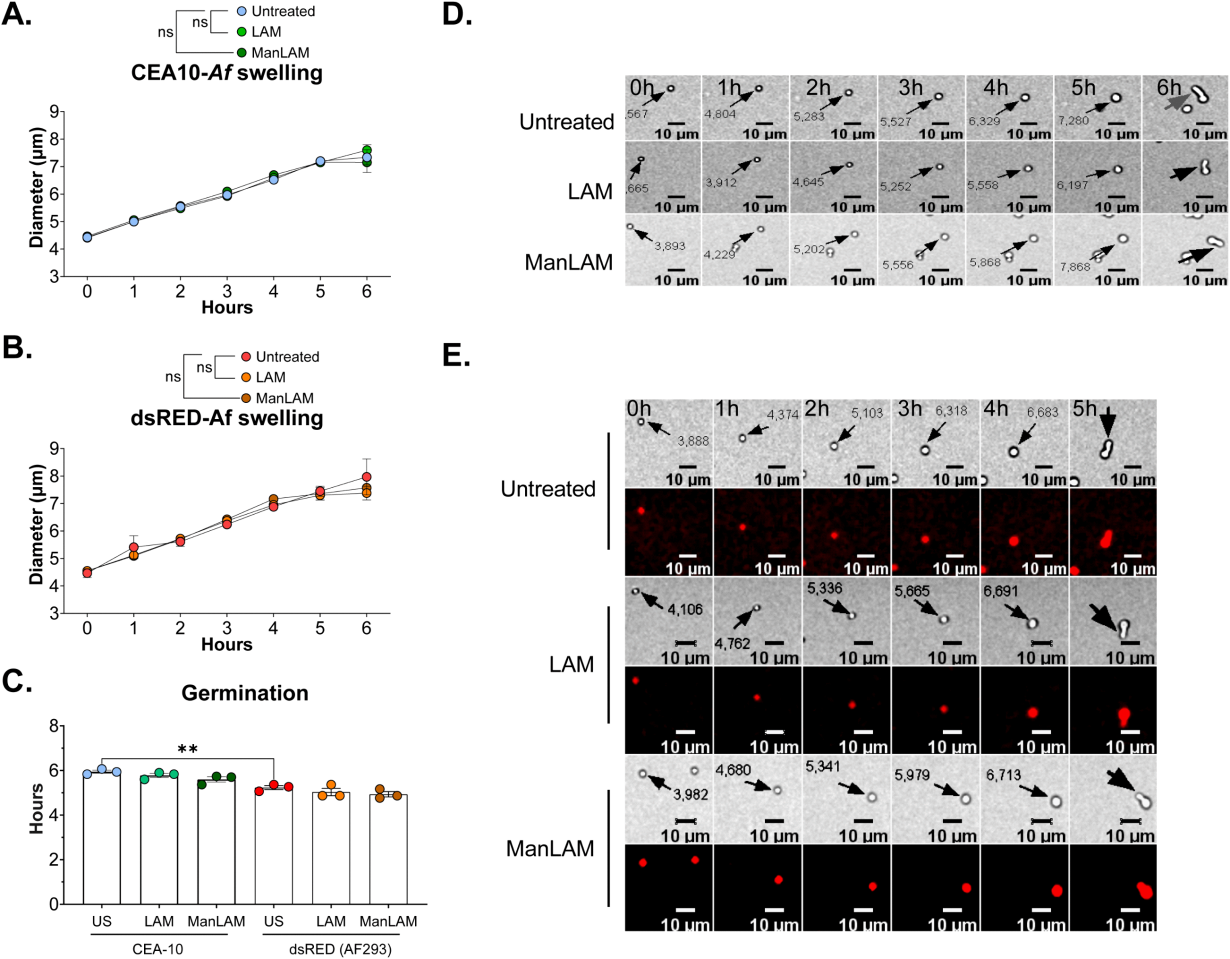
Lipoarabinomannan does not interfere with *Af* fitness. **A.** Conidial diameter of CEA-10 *Af* strain over time. **B.** Conidal diameter for ds-RED *Af*. **C.** Comparison of the time of incubation at 37°C + 5% CO_2_ required to reach germination. **D.** Representative images of CEA-10 strain swelling until it reaches germination. **E.** Representative images of dsRED strain swelling and germination. Statistically significant differences were tested with two-way ANOVA for time-lapse microscopy experiment and one-way ANOVA for germination data. Statistical tests were performed on results from 3 experimental replicates (n = 3). Error bars in SEM. p value * < 0.05, ** < 0.01, *** < 0.001, **** < 0.0001.

We investigated if lipoarabinomannan alters conidial swelling and the incubation time required for germination. No statistically significant differences were found between conditions. Lipoarabinomannan did not change the swelling rate or time of incubation for germination. Mean diameter for untreated CEA-10 conidia was 4.4 µm at 0 hours and reached 7.3 µm by 6 hours. For dsRED-*Af*, the diameter ranged from 4.5 µm at 0 hours to 7.9 µm at 6 hours (Fig 1A and B).

For CEA-10, germination started approximately at 5.9 hours of incubation. LAM stimulation averaged 5.8 hours, and ManLAM 5.6 hours. Similar results were obtained with dsRED-*Af*, with untreated conidia reaching germination stage at 5.2 hours. For LAM, germination time-point was reached at 5 hours, and for ManLAM at 4.9 hours. These were not statistically significant differences (Fig 1C-E).

### LAM increases cell death in BMDMs infected with *Af*

**Fig 2:**
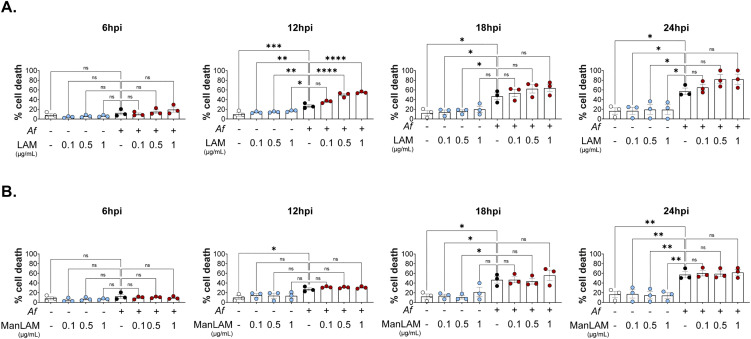
LAM increases cell death in *Af* infection. **A.** Cytotoxicity at 6, 12, 18 and 24 hpi measured as LDH release. Three concentrations of LAM (0.1 µg/mL, 0.5 µg/mL, and 1 µg/mL) were tested in uninfected controls (blue) and infected with swollen conidia of CEA-10 *Af* (red). **B.** Cytotoxicity at 6, 12, and 24 hpi with ManLAM at 0.1 µg/mL, 0.5 µg/mL, and 1 µg/mL. One-way ANOVA was performed on biological replicates (n = 3). Error bars in SEM. p value * < 0.05, ** < 0.01, *** < 0.001, **** < 0.0001.

LAM showed to increase cell death in BMDMs in a dose-dependent manner by 12 hpi. Mean values for cell death were 27%, 35.9%, 49.9%, and 54.9% for unstimulated, 0.1 µg/mL, 0.5 µg/mL, and 1 µg/mL of LAM respectively. The statistically significant difference is lost by 18 hpi; however, a clear trend persists with higher mean values for macrophages stimulated with LAM (Fig 2A). ManLAM did not produce any statistically significant differences (Fig 2B).

### LAM increases IL-1β release from *Af* infected BMDMs

**Fig 3:**
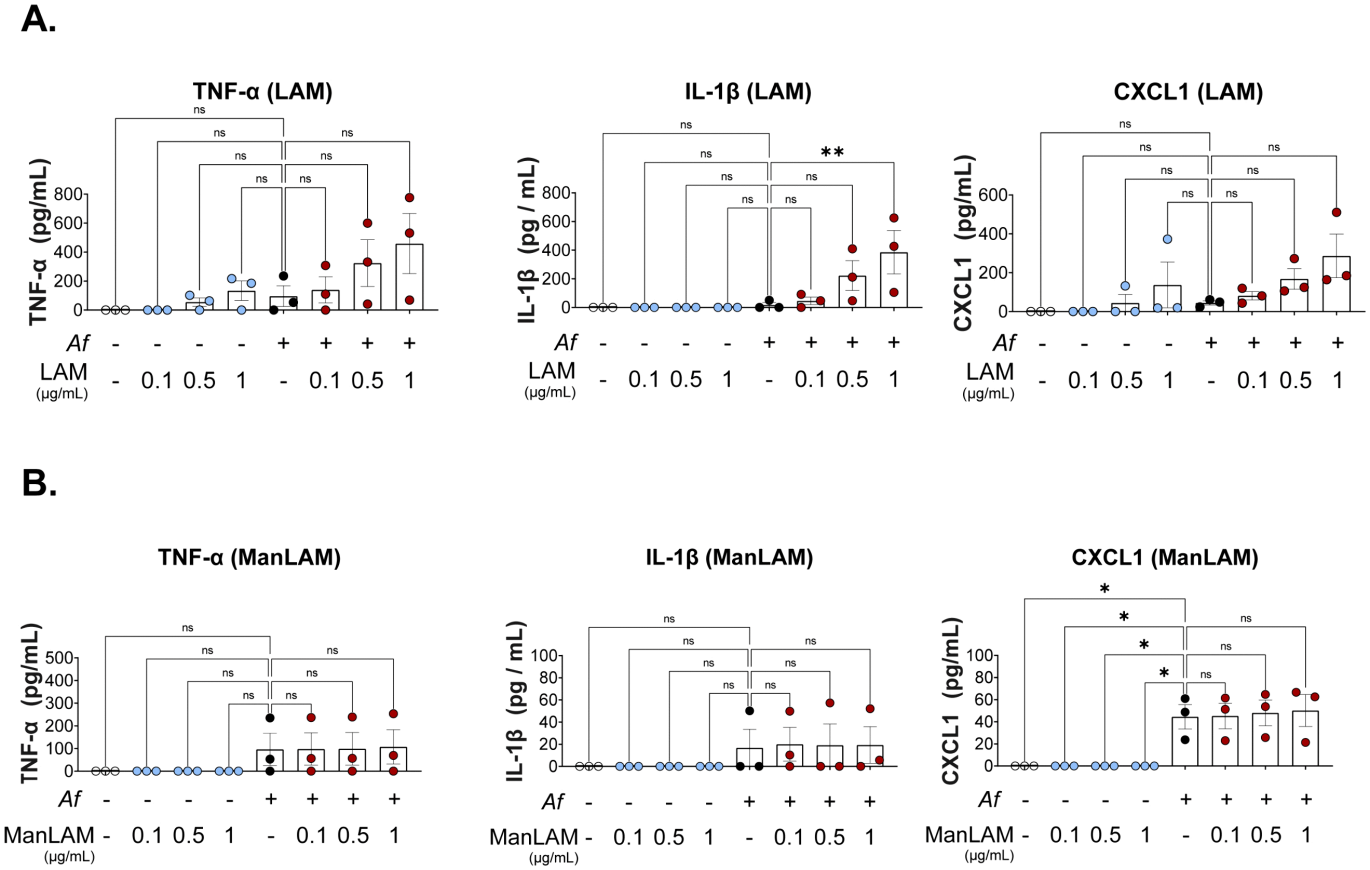
LAM increases IL-1β release with *Af* infection. **A.** Inflammatory cytokine release from BMDMs stimulated with different concentrations of LAM at 24 hpi. **B.** Inflammatory cytokine release from BMDMs stimulated with different concentrations of ManLAM at 24 hpi. One-way ANOVA was performed on biological replicates (n = 3). Error bars in SEM. p value * < 0.05, ** < 0.01.

BMDMs stimulated with LAM produced a significant increase of IL-1β release. Mean value for cells stimulated with 1 µg/mL of LAM was 385.8 pg/mL, whereas for unstimulated cells was 16.71 pg/mL (Fig 3A). It is important to note that at 24 hpi, more than 80% of LAM-stimulated macrophages had died, but only 57% of unstimulated macrophages were dead at 24 hpi. However, the increasing trend in IL-1β is suggestive of a stronger inflammatory response. Although there was an increasing trend in CXCL1 with higher concentrations of LAM, it was not statistically significant. TNF-α did not show a statistically significant difference. Finally, ManLAM did not have any effect on BMDMs cytokine release (Fig 3B).

### Mycobacterial lipoarabinomannan reduces BMDMs *Af* killing rate

**Fig 4:**
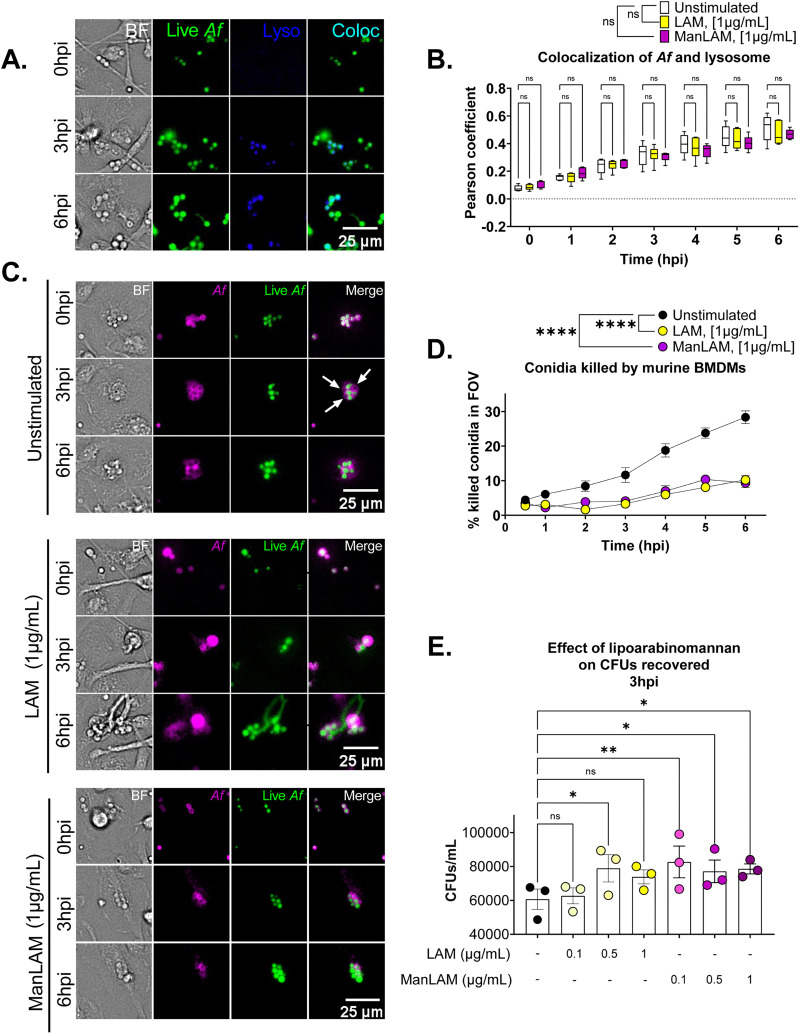
Mycobacterial ligands reduce macrophages killing rate of conidia. **A.** Representative images of colocalization analysis at 0, 3, and 6 hpi in unstimulated BMDMs infected with *Af*. **B.** Pearson coefficient for *Af*-lysosome colocalization over time show increasing trend over time for all conditions without statistically significant differences between them. **C.** Representative images of BMDMs killing *Af* conidia when unstimulated or treated with LAM or ManLAM. White arrows indicate dead Af conidia (single Alexa Fluor 633 positive). **D.** Percentage of killed conidia (counted as single Alexa Fluor 633 positive signal) over time for unstimulated, LAM, and ManLAM treated BMDMs. **E.** CFUs results from *Af* infected BMDMs either unstimulated or stimulated with either LAM or ManLAM at 3 hpi. Two-way ANOVA test for colocalization experiment and conidia killing assay. Box-plot centre line, median; + , mean, upper and lower quartiles; whiskers, 10^th^ and 90^th^ percentile. One-way ANOVA for CFUs. Statistical significance was tested on biological replicates (n = 3). p value * < 0.05, ** < 0.01, *** < 0.001, **** < 0.0001.

Next, we studied the effect 1 μg/mL for LAM and ManLAM on lysosome delivery and conidia killing. Pixels colocalization between LysotrackerBlue and dsRED-expressing conidia showed no statistically significant differences between lipoarabinomannan and unstimulated cells (Fig 4A and B).

Nonetheless, using a FLuorescent Aspergillus REporter (FLARE) conidia [28], which allows the tracking in real-time of fungus clearance, a significant decrease in conidia killing was detected with both, LAM and ManLAM. By 2 hpi, LAM killed 1.7% vs 8.4% from unstimulated controls, which represents 80% less killing. At 6 hpi, LAM killed 10.2% of conidia vs 28.3% from unstimulated cells, or 74% less conidia. For ManLAM, the effect was similar. By 1 hpi, ManLAM cleared 2.5% vs 6.1% in controls. At 6 hpi, BMDMs stimulated with ManLAM cleared only 9.4% of conidia (Fig 4C and D). These findings were confirmed with CFUs counting. At 3 hpi, significant more CFUs were obtained from cells stimulated with LAM and ManLAM. LAM showed a dose effect, with statistically significant difference detected at 0.5 μg/mL but lost at 1 μg/mL. Similarly, ManLAM showed a stronger effect with 0.1 μg/mL than with 0.5 and 1 μg/mL (Fig 4E).

## Discussion

Here, we show that mycobacterial ligand, lipoarabinomannan, negatively modulates macrophage responses to *Af* conidia at early stages of infection. LAM increased cell death and IL-1β release, while LAM and ManLAM decreased the killing of *Af* conidia at early stages of infection in an equivalent manner. These results suggest that mycobacterial virulence factors can interfere effectively against early immune response towards *Af* spores.

*Af* adaptation to its environment and its metabolic versatility has been highlighted as a factor contributing to its success as an opportunistic pathogen [29]. Furthermore, interference with metabolic pathways has been shown to modify its virulence significantly. For example, high availability of glucose and acetate, carbon energy sources, has been shown to change cell-wall components that enhance the resistance to oxidative stress [30]. It is unknown if mycobacteria have the capacity to modulate fungal virulence. However, fungi have been shown to produce arabinases, which metabolizes sugars towards L-arabinose and the activation of the pentose-phosphate pathway [31]. Whether if lipoarabinomannan can biochemically modulate *Af* fitness or development via induction of specific metabolic pathways is uncertain. Nonetheless, we showed that neither LAM or ManLAM produced any changes in the growth rate and germination of *Af* conidia, which suggests that our findings are most likely consequence of the modulation of macrophage responses and not due to early adaptations on fungi fitness, interpreted as growth rate and conidiation capacity [9]. It should be noted that three strains of A. fumigatus were used across assays in this work due to different visualisation needs. It is known that there are differences in virulence between strains such as CEA10 and AF293, however we observed consistent trends unaffected by strain background, confirming that our findings reflect immune modulation rather than strain-specific effects.

BMDMs stimulation with LAM produced a significant increase in cell death. Mycobacteria have been shown to alter cell death pathways as a survival strategy [32,33]. We suggested that these survival strategies prime macrophages to respond abnormally to *Af*. When macrophages were stimulated with LAM, more cells died faster, releasing significantly more IL-1β. It should be noted that, particularly at later infection time points where substantial macrophage death was observed, extracellular IL-1β levels may partially reflect passive release from dying cells in addition to active inflammasome-associated secretion. Therefore, these findings should be interpreted in the context of the overall cytotoxic phenotype induced by LAM. *M. smegmatis*’ LAM is a strong TLR-2 agonist that leads to activation of NF-κβ. This has been shown to induce a hyperinflammatory response, characterised by increased expression of inflammasome components, and pyroptotic cell death [34,35]. Findings with LAM were also consistent in J774A.1 cells (S1 and S2 Fig), which showed increased cell death in a concentration-dependent manner and increased IL-1β release. This is consistent with NLRP3 inflammasome activation model [36], using LAM as signal 1 (priming), and *Af* as signal 2 (activation). Further investigation focused on the delivery of lysosome to the phagosome and conidia killing, since lipoarabinomannan has been shown to have the capacity to interfere with phagosome maturation and phago-lysosome fusion [37,38]. Both, LAM and ManLAM showed significant decrease in the capacity to kill conidia within the first 6 hours of infection. However, this finding did not correlate with a reduced colocalization of lysosomes with *Af*. A 3 hpi time-point was employed for counting CFUs from lysed macrophages, which corroborated the results using time-lapse microscopy. The absence of differences in lysosome colocalization despite impaired fungal killing suggests that LAM and ManLAM may affect antifungal effector functions downstream of lysosomal recruitment rather than phagosome trafficking itself. Consistent with this, Lysotracker staining did not reveal substantial alterations in phagosomal acidification between conditions. Therefore, altered macrophage antimicrobial activity, including defects in ROS production, phagolysosomal enzymatic activity, or other functional maturation processes, may contribute to the reduced killing phenotype observed. Further investigation will be required to define these mechanisms.

Although, ManLAM binds preferentially to mannose receptor in macrophages [39], it also binds and activates TLR-2, eliciting inflammatory cytokine release [40,41]. Interestingly, the stimulation of macrophages with 1 μg/mL of ManLAM led to reduced killing rate of conidia, without increased cell death or a differentiated inflammatory cytokine release. The distinct effects observed between LAM and ManLAM at equivalent concentrations likely reflect structural differences that influence receptor engagement and downstream signalling. The mannose cap present in ManLAM promotes interaction with mannose receptor-mediated pathways, whereas uncapped LAM is associated with stronger TLR-2-driven inflammatory signalling. Consequently, these glycolipids may differentially modulate macrophage activation states and antifungal effector functions despite being present at the same concentration. In addition, glycolipid-specific differences in ligand presentation, receptor affinity, signalling thresholds, or aggregation behaviour in solution may further contribute to the divergent phenotypes observed. Further studies are necessary to dissect these receptor-specific and biophysical mechanisms in greater detail. Of note, Higher concentrations of ManLAM, 10–20 µg/mL [42], have shown increased cytokine release, arrest of calcium-dependent processes, and apoptosis [43]. However, we avoided these concentrations since they would have masqueraded priming.

Future studies should aim to characterise more deeply the broader effects of LAM on fungal-driven inflammation kinetically, and the impact on cell exhaustion. The failure to kill *Af* conidia at early stages of infection suggests that these mechanisms might be compromised. To corroborate the clinical significance of these findings, further testing using human monocyte-derived macrophages from patients with *Mtb* and atypical mycobacterial infections would be beneficial.

We propose that mycobacterial virulence factors may alter the early immune response to conidia, particularly because inflammasome activation has primarily been reported in response to hyphae [19,21]. Lipoarabinomannan (LAM), a major component of the mycobacterial cell envelope, is recognized by several innate immune receptors, including TLR2, the mannose receptor, and C-type lectins [39,40]. Engagement of these receptors can modulate macrophage activation and inflammatory signalling, thereby potentially influencing subsequent responses to *A. fumigatus*. We therefore speculate that exposure to mycobacterial components may prime or alter macrophage inflammatory responses before or during interaction with *A. fumigatus*. Because signalling through these receptors can converge on NF-κB activation, mycobacterial-induced macrophage activation may influence inflammasome priming and downstream inflammatory responses. However, the precise mechanisms underlying this interaction were not investigated in the present study and require further experimental validation.

In conclusion, our results indicate that lipoarabinomannan can modulate macrophage responses to *Af*, interfering with clearance of fungi. LAM, lipoarabinomannan without mannose cap, induces a hyperinflammatory response characterised by increased release of IL-1β and increased rate of macrophages death.

## Supporting information

S1 Fig LAM increases cell death and IL-1β release in J774A.1 cells infected with *Af* A. Time-lapse fluorometric assay using PI as a cell death marker. Percentages were calculated as relative to control with 0.1% TX100. **B.** Concentration of TNF-α in supernatant from J774A.1 cells infected with *Af* for 6, 12, and 18 hpi. **C.** Concentration of IL-1β in supernatant from cells infected with *Af* for 6, 12, and 18 hpi. Both cytokines were measured simultaneously from the same sample. One-way ANOVA was performed on 3 experimental replicates (n = 3). Error bars in SEM.
(TIF)

S2 Fig LAM reduced phagolysosome fusion at early stages of infection in J774A.1 cells. A. Colocalization of eGFP-*Af* and Lysotracker™ Blue DND-22 at 1, 2, and 3 hpi presented as Pearson’ Coefficient. Samples pooled from 3 experimental replicates for n = 81 cells per condition. B. Representative images widefield time-lapse fluorescence microscopy showing J774A.1 cells, stained with CD45-AF700 (red) stimulated with 0.5 µg/mL of LAM infected with eGFP-*Af* (green) and stained for lysosome LysoTracker™ Blue DND-22 (blue). Colocalized areas can be identified in cyan. C. CFUs obtained from J774A.1 cells lysed at 3 hpi. Two-way ANOVA test for colocalization experiment and one-way ANOVA for CFUs. Box-plot centre line, median; + , mean, upper and lower quartiles; whiskers, 10^th^ and 90^th^ percentile.
(TIF)
